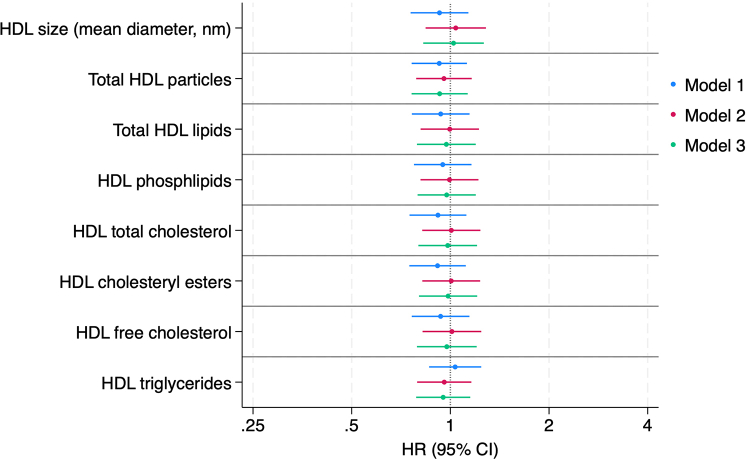# High-density lipoprotein subfractions and risk of future venous thromboembolism—the HUNT study

**DOI:** 10.1016/j.rpth.2025.103295

**Published:** 2025-12-09

**Authors:** Inga A. Røstvold, Ben Brumpton, Kristian Hveem, Bjørn Olav Åsvold, Guro F. Giskeødegård, George Davey Smith, Nicholas J. Timpson, Kaitlin H. Wade, John-Bjarne Hansen, Sigrid K. Brækkan

**Affiliations:** 1 Thrombosis Research Group (TREC), Department of Clinical Medicine, UiT – The Arctic University of Norway, Tromsø, Norway; 2 HUNT Center for Molecular and Clinical Epidemiology, Norwegian University of Science and Technology (NTNU), Trondheim, Norway; 3 Levanger Hospital, Nord-Trøndelag Hospital Trust, Levanger, Norway; 4 Clinic of Medicine, St. Olav’s Hospital, Trondheim University Hospital, Trondheim, Norway; 5 HUNT Research Centre, Department of Public Health and Nursing, Norwegian University of Science and Technology (NTNU), Levanger, Norway; 6 Department of Endocrinology, Clinic of Medicine, St. Olav’s Hospital, Trondheim University Hospital, Trondheim, Norway; 7 Department of Public Health and Nursing, Norwegian University of Science and Technology (NTNU), Trondheim, Norway; 8 Department of Surgery, St. Olavs University Hospital, Trondheim, Norway; 9 MRC Integrative Epidemiology Unit (IEU), Bristol Medical School, University of Bristol, Bristol, UK; 10 Thrombosis Research Centre (TREC), Division of Internal Medicine, University Hospital of North Norway, Tromsø, Norway

**Keywords:** cohort studies, deep vein thrombosis, high-density lipoprotein, pulmonary embolism, venous thrombosis

## Abstract

**Background:**

Previous studies on high-density lipoprotein (HDL) cholesterol levels and the risk of venous thromboembolism (VTE) have shown conflicting results, and it has been suggested that specific HDL subfractions and lipid composition may be more informative with regards to VTE risk.

**Objectives:**

We aimed to investigate the association between HDL subfractions (including particle size, concentration, and lipid composition) and risk of VTE in a population-based cohort.

**Methods:**

The study included 17,032 participants from the Trøndelag Health Study (HUNT3) cohort conducted in 2006-2008 who were followed up until December 31, 2019. HDL subfractions were analyzed in serum using nuclear magnetic resonance spectroscopy. All incident VTEs during follow-up were validated and recorded. Cox proportional hazards regression models estimated hazard ratios (HRs) for the association between HDL metrics and incident VTE, adjusting for age, sex, and body mass index.

**Results:**

During a median follow-up of 12.0 years, 342 incident VTE cases were confirmed. No associations were found among HDL particle size, HDL concentration, HDL lipid composition, apolipoprotein (Apo)A1 levels, and VTE risk. All HRs per 1-SD increase in HDL metrics were within the range of 0.83 to 1.16 and had 95% CIs that included 1. Furthermore, quartile analyses of HDL particles (Q4 vs Q1—HR, 0.89; 95% CI, 0.65-1.21) and ApoA1 (Q4 vs Q1—HR, 0.94; 95% CI, 0.68-1.29) showed no associations with VTE risk.

**Conclusion:**

HDL subfractions, including particle size, concentration, lipid composition, and ApoA1, were not associated with the risk of a first-lifetime VTE event.

## Essentials

• High-density lipoprotein (HDL) subfractions may affect venous thromboembolism (VTE) risk.
• This study analyzed 17,032 participants from the HUNT3 cohort over 12 years.
• HDL subfractions showed no association with first-lifetime VTE risk.
• Findings suggest that HDL metrics may not play a role in the pathogenesis of VTE.

## 1 Introduction

Venous thromboembolism (VTE), consisting of deep vein thrombosis (DVT) and pulmonary embolism (PE), is a multifactorial disease affecting approximately 10 million people worldwide annually [1,2]. A substantial proportion of patients with VTE develop debilitating long-term complications [[3], [4], [5]], and the 1-year all-cause mortality after VTE is high [6,7]. While the incidence of arterial cardiovascular diseases (CVDs) has decreased markedly the last decades [8], the incidence of VTE has increased slightly in the same period [9]. The pathophysiologic mechanisms of VTE remain poorly understood, and risk assessment at the population level is challenging [1]. Identification of modifiable biomarkers involved in the pathogenesis of VTE may improve risk assessment and facilitate development of targeted therapies.

High-density lipoproteins (HDLs) are circulating particles composed of proteins, cholesterol, and various lipids. In addition to the reverse cholesterol transport and anti-inflammatory effects [10], HDL may exert antithrombotic properties by influencing platelet activation and endothelial function [11,12], enhancing the anticoagulant activity of protein C and protein S [13], as well as regulating fibrinolysis [12,14]. Previous studies on the relationship between HDL-cholesterol (HDL-C) and risk of VTE have reported somewhat conflicting results. A meta-analysis from 2008 [15], based on 4 case-control studies and 1 cohort study, found an inverse relationship between HDL-C levels and VTE risk. In contrast, 2 later comprehensive meta-analyses of 8 and 57 population-based cohorts published in 2016 [16] and 2019 [17], respectively, reported no association between HDL-C and VTE. Since plasma HDL particles vary substantially in size, lipid composition, and biological function, it has been suggested that specific HDL subfractions, rather than overall HDL-C, may be more informative for elucidating the role of HDL in the pathogenesis of VTE [11,13,18,19]. Interestingly, studies have suggested diverse antithrombotic properties across HDL subfractions. Larger HDL particles showed stronger inhibitory effect on platelet aggregation [10,20], and an inverse relationship was reported between HDL size and plasminogen activator inhibitor 1 levels, suggesting that larger HDL particles may enhance fibrinolysis [11]. On the contrary, smaller HDL particles were more closely linked with the endogenous anticoagulant tissue factor pathway inhibitor [10,21] and showed greater efficacy in preventing production of thromboxane B2 in human platelets, suggesting an antithrombotic effect [11].

While large meta-analyses indicate that total HDL-C levels are not linked to VTE risk [16,17,22], there is limited knowledge on whether differences in HDL particle size and lipid composition relate to VTE. A small case-control study (49 vs 49) in young men (<55 years) reported a higher prevalence of large HDL particles in controls than that in VTE cases, suggesting a protective effect of large HDL particles on VTE [23]. Furthermore, a recent study using data from a subset of the UK Biobank (*n* = 95,402) showed that larger HDL particles are associated with lower risk of VTE, but Mendelian randomization analyses did not support a causal relationship [24]. Of note, the analyses were not adjusted for body mass index (BMI), a potentially important confounder for the observational study [24], as increasing BMI is associated with lower HDL-C levels, altered HDL subfraction distribution, and increased risk of VTE [[24], [25], [26], [27]]. In the present study, we aimed to comprehensively investigate the association between HDL subfractions (particle size, particle concentration, and lipid composition) and risk of future VTE using a population-based cohort, adjusting for appropriate confounding factors.

## 2 Methods

### 2.1 Study population and design

This cohort study is based on data from the third survey of the Trøndelag Health Study, HUNT3. The HUNT study is a Norwegian follow-up study of residents of the former Nord-Trøndelag County [28]. In HUNT3, conducted between August 2006 and June 2008, all inhabitants aged 20 years or older were invited to participate. The survey gathered information through biological samples, questionnaires, interviews, and clinical measurements. Of the 93,860 residents invited, 50,807 participated, resulting in a participation rate of 54.1%. Serum samples were collected at the time of inclusion and stored in a biobank [28]. This study included an unselected subsample of 17,215 individuals who participated in HUNT3 from May 2007 to June 2008 in whom metabolomics measurements was performed. Participants who moved from the county before inclusion (*n* = 6), those with known previous VTE (*n* = 118), and those with missing BMI values (*n* = 59) were excluded. Following these exclusions, the final cohort comprised 17,032 individuals. The study participants were followed from date of enrolment to December 31, 2019, and all VTE events during follow-up were validated and recorded. Participants who died or migrated were censored from the date of death or migration. All participants in HUNT3 provided written informed consent, and this study was approved by the Regional Committee for Medical and Health Research Ethics.

### 2.2 HUNT3 data and blood samples

At the time of inclusion, participants’ height (in centimeters) and weight (in kilograms) were measured objectively, and BMI was calculated, as previously described [28]. Information on smoking status, prevalent CVDs, and cancer history was collected through questionnaires. Prevalent CVD included a self-reported history of myocardial infarction, ischemic stroke, or angina pectoris. Nonfasting blood samples were collected at baseline inclusion and stored in the HUNT Biobank. Samples were drawn into serum separation tubes, left at room temperature for 30 to 90 minutes, centrifuged and transported at 4 °C to the biobank. Samples were fractionated the next day into 2D Matrix tubes (Matrix 2D; Thermo Fisher Scientific) and stored at −80 °C. The biobank uses continuous temperature monitoring with alarms and keeps logs for the refrigerator and transport cooler to ensure optimal storage. Detailed methodologies for blood sample collection and storage are outlined in the study by Næss et al [29].

In 2019, these samples were sent to the Nightingale Metabolomics Research Centre for high-throughput nuclear magnetic resonance (NMR) analysis. This technique enables both qualitative and quantitative assessments of metabolites, making it suitable for large-scale research, as demonstrated by several studies [[30], [31], [32], [33]]. In this study, NMR measurements were used to analyze the lipid composition and concentration of each HDL subfraction, defined by their average diameter: very large HDL (14.3 nm), large HDL (12.1 nm), medium HDL (10.9 nm), and small HDL (8.7 nm). Additionally, the concentration (in millimoles per liter) of total lipids in HDL, including phospholipids, total cholesterol, cholesteryl esters, free cholesterol, and triglycerides, were assessed. The concentration (in grams per liter) of apolipoprotein (Apo)A1 was also measured. A detailed list of the HDL subfractions is available in Supplementary Table 1.

### 2.3 Outcome assessment

The primary outcome was first-lifetime VTE. All first-lifetime events of DVT and PE were identified through the hospital discharge diagnosis registries at the hospitals covering the HUNT population (Levanger, Namsos, and St. Olavs Hospital) by an extensive search in International Classification of diseases, 10th Revision, codes related to VTE from 2006 to 2019. The medical records for each potential VTE case identified in these registries were reviewed by trained personnel, and data were extracted using a standardized form. A VTE diagnosis was adjudicated when symptoms of DVT or PE were confirmed by objective radiological procedures (such as ultrasound, venography, or computed tomography or through autopsy), and treatment was initiated (unless contraindications were present). In cases where a patient simultaneously had DVT and PE, the outcome was classified as PE.

A VTE event was classified as provoked if a provoking factor was present within 3 months prior to the event. Information on provoking factors was collected from the medical records and included active cancer, surgery, trauma, acute medical conditions (eg, ischemic stroke, acute myocardial infarction, severe infection), immobilization, or any factor explicitly noted as a provoking factor in the medical record (eg, intravascular catheter). Immobilization was defined as confinement to bed for 3 days or more, the need for a wheelchair, or the use of orthopedic cast.

### 2.4 Statistical analyses

All statistical analyses were carried out with STATA version 18.0 (Stata Corporation). Baseline characteristics were reported as means for continuous variables and as percentages for categorical variables. The distribution of total number of HDL particles, mean HDL particle size and lipid composition in HDL subfractions was displayed using descriptive statistics. The correlation between BMI and mean HDL particle size was displayed in a scatter plot, and the Pearson correlation coefficient was estimated. The person-time of follow-up for each participant was determined from the inclusion date in HUNT3 until the earliest of the following events: date of a first VTE, migration from the county, death, or end of the study period (December 31, 2019). Incidence rates were calculated by dividing the number of events by the total person-time and expressed as events per 1000 person-years. Cox proportional hazards regression models, with age as the time scale, were performed to estimate hazard ratios (HRs) with 95% CIs for the associations between HDL particle concentration, total HDL lipids, HDL subfractions (particle size, concentration, and lipid composition), ApoA1 concentration, and the risk of incident VTE. Adjustments were made for potential confounders using 3 models: model 1 included age and sex; model 2 included age, sex, and BMI; and model 3 included age, sex, BMI, and CVD. All HDL subfraction variables were analyzed as continuous variables, with HRs expressed per SD increase. A quartile analysis was performed to investigate the proportion of very large and combined very large + large HDL particles and the risk of incident VTE. Additionally, a quartile analysis of ApoA1 concentration, total HDL particle concentration, and risk of VTE was conducted. In the quartile analyses, the variables were divided into 4 quartiles, using the lowest as the reference group. The proportional hazards assumption for the Cox models was tested using Schoenfeld residuals. A linear regression analysis was performed to investigate the correlation between total HDL particles and ApoA1 concentrations. We additionally performed a subgroup analysis assessing the association between HDL particle characteristics and risk of unprovoked VTE.

Post hoc power calculations demonstrated that our study, comprising 17,032 participants and 342 VTE events, had 80% power at a 2-sided significance level of α = 0.05 to detect an HR of 0.86 or 1.16 per 1-SD change in HDL measures, corresponding to a 14% to 16% change in hazard. This indicates sufficient power to detect clinically meaningful differences in VTE risk across HDL phenotypes. Since our study population consisted of a random subsample from HUNT3, we assessed the association between well-established risk factors (age and BMI) and VTE, to ensure that expected associations were detected in our population (positive controls).

## 3 Results

The baseline characteristics of the 17,032 participants included in the study are presented in Table 1. The average age was 52.2 years, the mean BMI was 27.1 kg/m^2^, and 46% were men. During a median follow-up of 12.0 years (range, 0.04-12.6 years), 342 symptomatic incident VTE events were confirmed. Details of the VTE events are provided in Table 2. The average age at first-lifetime VTE was 69.5 years, with 53.2% of the cases occurring in men. Among the VTE events, 65.5% were provoked, and 55.6% presented as PE.

**Table 1:** Baseline characteristics of the study population.

Baseline characteristics (*N* = 17,032)	Value
Age (y)	52.2 ± 15.7
Sex: male	46.0 (7837)
BMI (kg/m^2^)	27.1 ± 4.3
Cancer	5.0 (849)
CVD	7.2 (1217)
Smoking status: daily	16.9 (2885)

Continuous variables are shown as mean ± SD. Categorical variables are shown as % (*n*). CVD included a history of myocardial infarction, stroke, or angina pectoris.

BMI, body mass index; CVD, cardiovascular disease.

**Table 2:** Characteristics of the venous thromboembolism (VTE) events (*n* = 342).

Characteristic	Value
Age at VTE (y)	69.5 ± 13.6
Sex (male)	53.2 (182)
Deep vein thrombosis	44.4 (152)
Pulmonary embolism	55.6 (190)
Unprovoked VTE	34.5 (118)
Provoked VTE	65.5 (224)
Cancer	26.3 (90)
Surgery	25.7 (88)
Immobilization	25.4 (87)
Trauma	17.5 (60)
Other factors	5.0 (17)
Acute medical conditions	4.7 (16)

Age is shown as mean ± SD, and categorical variables as % (*n*).

To confirm that well-established risk factors for VTE showed expected associations in our study population, we examined the associations of age and BMI with risk of VTE. Age was significantly associated with increased VTE risk, with an HR of 1.72 (95% CI, 1.60-1.86; *P* < .001) per 10-year increase in age. BMI was also positively associated with VTE risk (HR, 1.20; 95% CI, 1.12-1.28; *P* < .001) per 3-unit increase. These associations are consistent with previously reported relationships between age, BMI, and VTE [1,15], supporting the validity of the present dataset.

**Figure 1:**
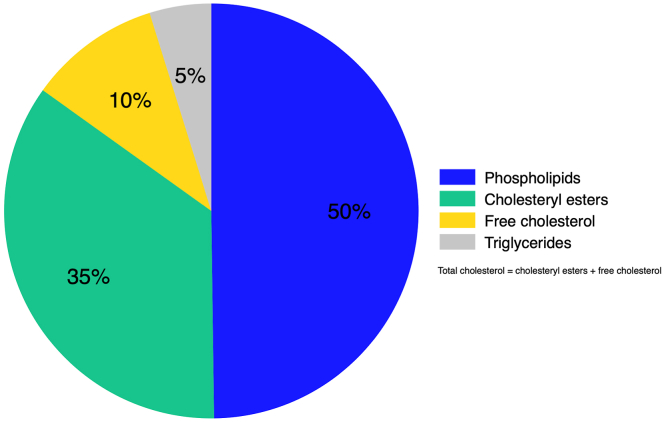
Average lipid composition in high-density lipoprotein (HDL). Pie chart of the mean percentage distribution of lipids within HDL among the study population.

**Figure 2:**
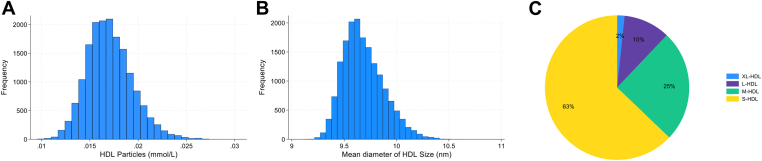
Characteristics of high-density lipoprotein (HDL) particles in the study population. (A) Distribution of total HDL particle concentrations (mmol/L). (B) Distribution of average HDL diameter sizes (nm). (C) Pie chart depicting the average distribution of HDL subfractions, categorized as very large (XL), large (L), medium (M), and small (S) HDL, based on mean concentrations (mmol/L).

**Supplementary Figure 1:**
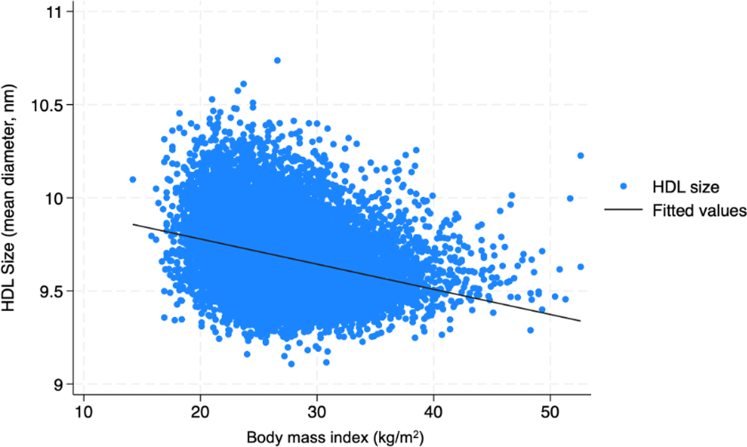


The average distribution of lipids within HDL particles (total HDL) were 50% phospholipids, 35% cholesteryl esters, 10% free cholesterol, and 5% triglycerides (Figure 1). Figure 2 shows the overall distribution of HDL particles (mean concentration and mean size) and HDL subfractions in the study population. The overall mean concentration of HDL particles in the study population was 17.05 μmol/L (Figure 2A). The histogram of average HDL size showed a right-skewed distribution, as most participants had HDL particles with a smaller size (Figure 2B). The distribution of subfractions showed 63% small, 25% medium, 10% large, and 2% very large HDL particles (Figure 2C). The mean concentration of small HDL particles was 10.72 μmol/L, while the mean concentration of very large HDL particles was 0.28 μmol/L (Supplementary Table 1). There was an inverse correlation between BMI and mean HDL particle size (*r* = −0.30; *P* < .001) (Supplementary Figure 1).

**Figure 3:**
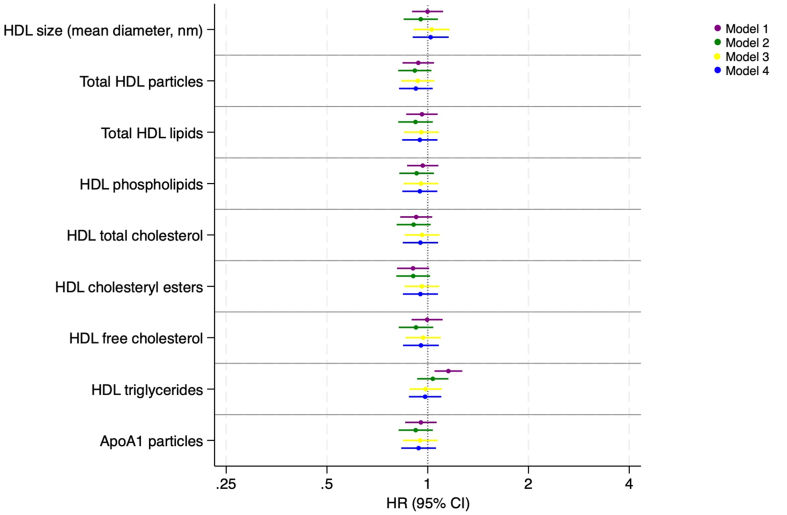
Association between high-density lipoprotein (HDL) particle characteristics and risk of incident VTE. Forest plot illustrating the hazard ratios (HRs) for venous thromboembolism associated with HDL particle characteristics, presented with 95% CI per 1-SD increase. The analysis includes 4 models. Model 1 is unadjusted. Model 2 is adjusted for age and sex. Model 3 is adjusted for age, sex, and body mass index (BMI). Model 4 is adjusted for age, sex, BMI, and cardiovascular disease.

**Supplementary Figure 2:**
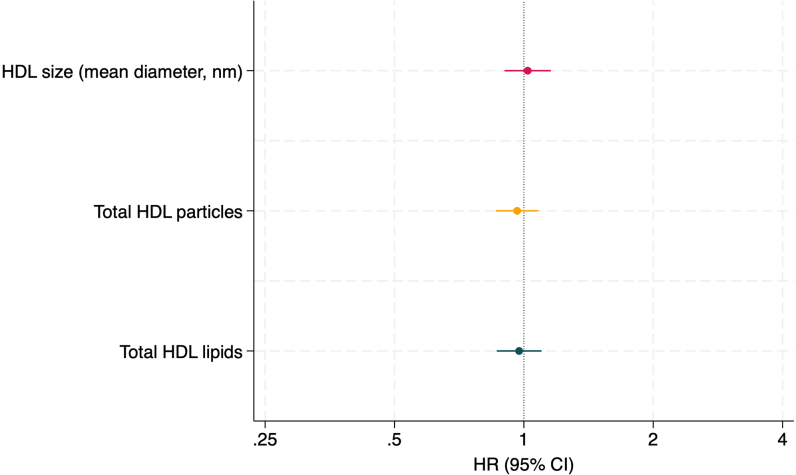


The HRs of VTE according to overall measures of HDL size (in nanometers), HDL particle concentration (in millimoles per liter), and total HDL lipids (in millimoles per liter) are presented in Figure 3. In the model adjusted for age, sex, and BMI (model 3), there were no associations between 1-SD increase in any of these HDL metrics, and the risk of incident VTE, as all HRs were within the range 0.833 to 1.162 with 95% CIs that overlapped the null. Further adjustment for CVD did not alter the results (model 4). Similarly, in sensitivity analyses with follow-up restricted to the first 5 years, there was no association between HDL size (in nanometers), HDL particle concentration (in millimoles per liter), and total HDL lipids (in millimoles per liter) and risk of VTE (Supplementary Figure 2). Furthermore, subgroup analysis with unprovoked VTE as the outcome showed no associations between the total HDL parameters and unprovoked VTE (Supplementary Figure 6).

**Figure 4:**
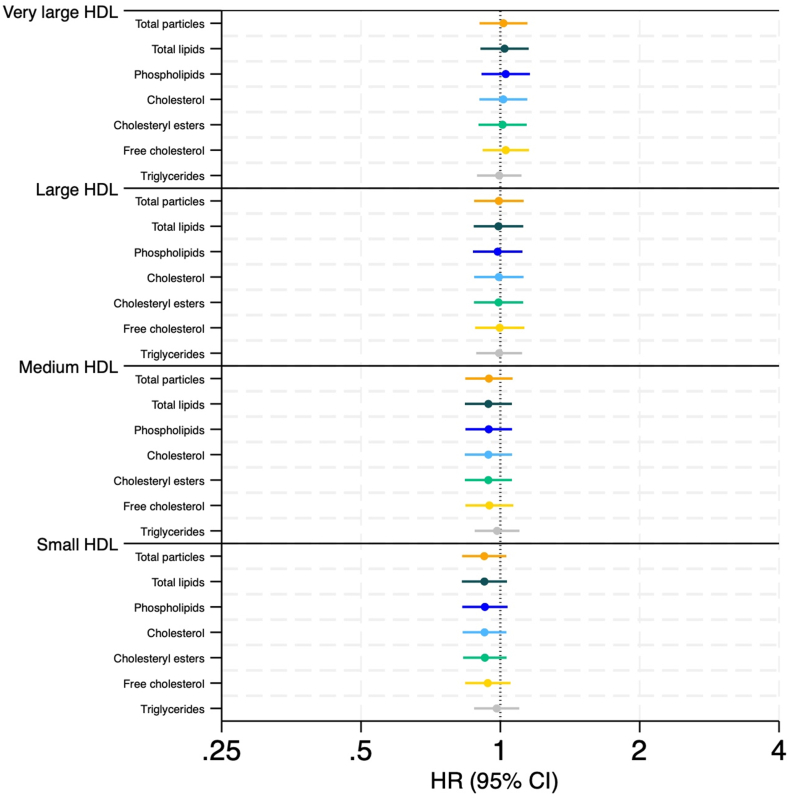
Association between lipid composition of high-density lipoprotein (HDL) subfractions and risk of incident venous thromboembolism (VTE). Forest plot presenting hazard ratios (HRs) for VTE in relation to lipid composition and total concentration of HDL subfractions. Each HR is presented with a 95% CI per 1-SD increase. The analysis is adjusted for age, sex, and body mass index.

Supplementary Figure 4 depicts the mean lipid concentration (in millimoles per liter) of total cholesterol, cholesteryl esters, free cholesterol, phospholipids, and triglycerides in each HDL subfraction, reflecting the greater prevalence of small HDL lipids than that of very large HDL lipids. The HRs of VTE according to measures of HDL subfractions and their total lipid concentration and lipid composition (in millimoles per liter) are presented in Figure 4. In the model adjusted for age, sex, and BMI, no statistically significant associations were found between 1-SD increase in any of these HDL metrics and the risk of incident VTE. The HRs ranged from 0.826 to 1.158, with CIs that overlapped the null (Figure 4).

As expected, there was a strong correlation (*r*^2^ = 0.90) between measured ApoA1 and total HDL particles (Supplementary Figure 5A). Quartile-based analyses of ApoA1 (Q4 vs Q1—HR, 0.94; 95% CI, 0.68-1.29) and total HDL particles (Q4 vs Q1—HR, 0.89; 95% CI, 0.65-1.21) similarly provided limited evidence for an association with risk of VTE (Supplementary Figure 5B).

**Supplementary Figure 3:**
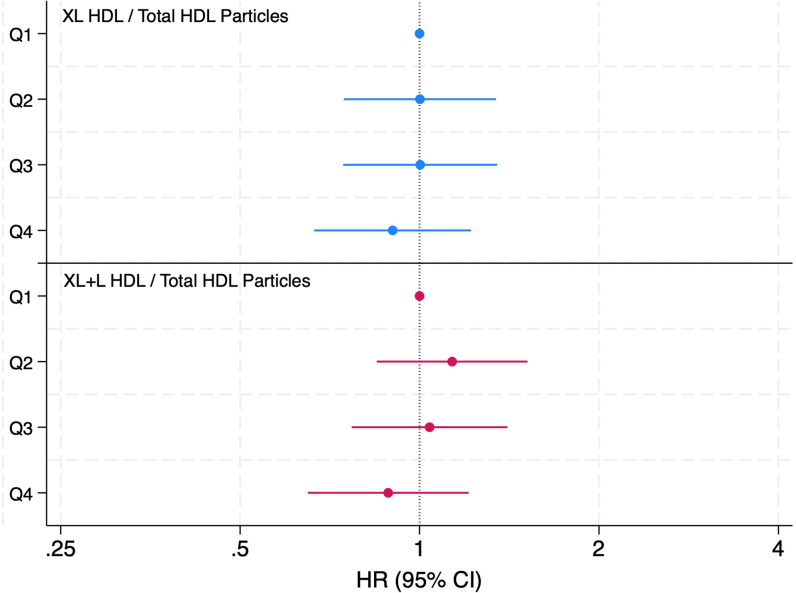


**Supplementary Figure 4:**
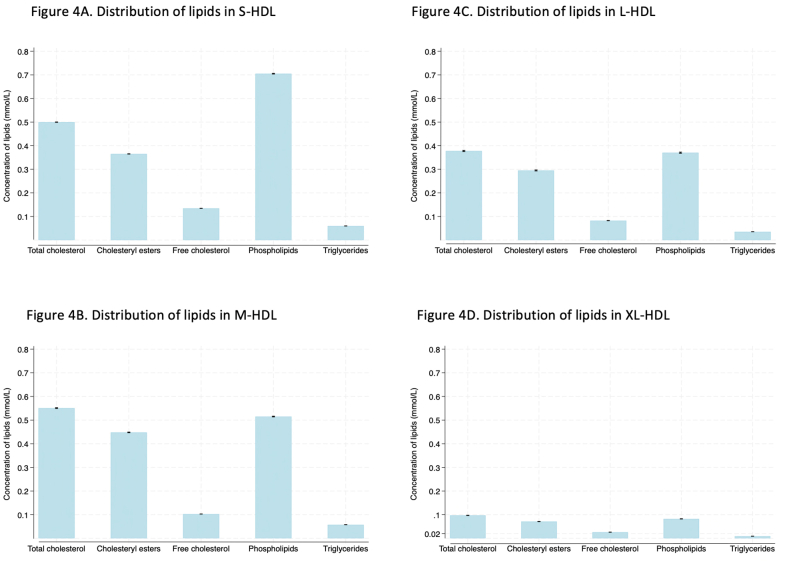


**Supplementary Figure 5:**
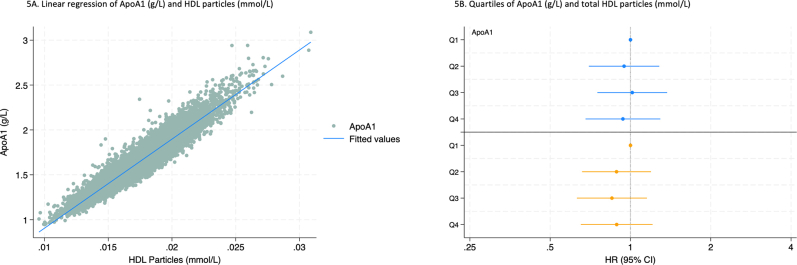


Quartile analysis of the proportion of larger HDL particles and the risk of incident VTE are presented in Supplementary Figure 3. Increasing proportion of very large HDL particles showed no association with VTE risk, with an HR of 0.90 (95% CI, 0.67-1.22) for the highest vs lowest quartile. Similarly, increasing proportions of large and very large HDL particles were not associated with VTE, with an HR of 0.89 (95% CI, 0.65-1.21) for Q4 vs Q1 in the adjusted model.

## 4 Discussion

This population-based cohort investigated the association between HDL subfractions (particle size, concentration, and lipid composition), ApoA1, and the risk of future venous thromboembolism. On average, the lipid content in HDL comprised 50% phospholipids, 35% cholesteryl esters, 10% free cholesterol, and 5% triglycerides. The majority of the HDL particles were small (63%), and the proportion of very large particles was low (2%). There was little evidence for a strong association between any of the examined HDL parameters and the risk of first-life time VTE. Our findings suggest that HDL is not involved in the pathogenesis of VTE.

The distribution of HDL subfraction sizes in our study population aligned well with previous findings from the UK Biobank cohort [34]. The median HDL size in our cohort was 9.656 nm (IQR, 9.542-9.804 nm), while it was 9.609 nm (IQR, 9.490-9.771 nm) in the UK Biobank. Furthermore, the median and IQR for total HDL lipids was slightly higher in our cohort (3.280 mmol/L; IQR, 2.883-3.737 mmol/L) than those for the UK Biobank (2.905 mmol/L; IQR, 2.522-3.359 mmol/L). This difference could presumably be explained by the lower median age at recruitment in our cohort (53 years; IQR, 41-63 years) than that of the UK Biobank (58 years; IQR, 50-63 years), as HDL-C has been shown to decrease with age [34,35].

A small case-control study by Deguchi et al. [23] reported lower plasma concentrations of total HDL particles, large HDL particles, HDL-C, and ApoA1 in patients with VTE than those of controls [23]. This study was limited to men with unprovoked VTE (<55 years), which could affect the generalizability of the results. Furthermore, the control group was sourced from a blood donation program, which could introduce bias due to the healthy donor effect [36], and blood samples were collected after the VTE event, which could lead to reverse causation as HDL-C and ApoA1 levels has been shown to decrease in the acute phase after VTE [10,[37], [38], [39]]. In contrast to the findings by Deguchi et al. [23], and in agreement with our findings, a study by Hald et al. [40] found no differences in HDL sizes between 20 patients with unprovoked VTE and 20 matched controls. A study by Lee et al. involving 95,402 UK Biobank participants suggested a minor protective effect of increasing HDL size, total HDL lipids, HDL-C, and specific HDL lipid components on VTE risk [24], whereas a higher proportion of triglycerides in HDL subfractions was associated with an increased risk of VTE. However, Mendelian randomization analysis did not support that these relationships were causal [24]. Consistently, a recent Mendelian randomization study of 6 cohorts found no causal relationship between HDL-C levels and VTE risk [22]. The analyses of HDL subfractions in the study by Lee et al. [24] were not adjusted for BMI, which is likely an important confounder. HDL-C is known to decrease with increasing BMI [25,26], and increasing BMI is associated with increased risk of VTE [15,[41], [42], [43]]. In the MEGA case-control study, Morelli et al. [39] also found an association between low HDL-C levels and increased VTE risk, but the association was attenuated and no longer statistically significant after adjusting for BMI. In the present study, we additionally showed an inverse correlation between BMI and HDL particle size, and the risk estimates for the HDL parameters on VTE were attenuated after adjustment for BMI, emphasizing the importance of this confounder.

Previous studies on the association between HDL-C and VTE have shown diverging results. A meta-analysis by Ageno et al. [15], comprising 4 case-control studies and 1 cohort study, found an inverse relationship between HDL-C levels and VTE risk [15]. Similarly, Mi et al. [44] found lower HDL-C levels in VTE patients across 5 case-control and 4 cohort studies. However, heterogeneity among the included studies, lack of adjustment for important confounders (BMI), and potential biases (reverse causation and control group selection), complicated the interpretation of these results [15,44]. In a more recent and larger meta-analysis, Gregson et al. [17] found no association between HDL-C levels and VTE risk across 57 cohorts, although they noted an inverse association with ApoA1 (HR, 0.65; 95% CI, 0.47-0.88) in a subset of 20 cohort studies. The meta-analysis included only fatal VTE events, and participants were selected based on criteria such as the absence of CVD, which may limit its generalizability [17]. A meta-analysis of 9 population-based cohorts with symptomatic and objectively verified VTE events found no association between HDL-C and VTE [16]. The included cohorts had individual participant-level data of high quality, including objective assessment of BMI, and achieved the maximum score on the Newcastle-Ottawa Quality Assessment Scale for cohort studies [16].

Strengths of this study include the large number of participants drawn from a general population, the long follow-up period, validated VTE events and thorough assessment of BMI as an important confounder, which enhance internal validity and reduce potential bias. Blood samples were collected prior to the VTE event and were securely stored in a biobank, preserving the temporal sequence between exposure and outcome. The study adjusted for important confounders, including age, sex, and BMI. Metabolites identified through NMR have been validated using multiple analytical techniques, including gas chromatography, routine clinical chemistry assays, mass spectrometry, and enzymatic methods, ensuring data accuracy and reliability [30,[45], [46], [47]]. The capability of NMR to measure HDL subfractions directly from serum without preliminary isolation further enhances data reliability [48]. A potential limitation was the use of nonfasting blood samples. However, existing research suggests that fasting and nonfasting HDL-C levels do not differ significantly, and only minor changes in HDL size are observed postprandially [40,49]. We would therefore not expect large variations in the other HDL parameters. Any variability due to the nonfasting status would be nondifferential, as the composition of, and time since, the last meal at blood draw would likely be random. Furthermore, any misclassification of HDL parameters would not be influenced by the outcome. Blood samples were collected only once at inclusion, and random fluctuations in HDL could introduce random misclassification over time. Any random misclassification could potentially lead to underestimating the true association between HDL and VTE risk. Nevertheless, when the follow-up period was restricted to the first 5 years, the HRs for VTE according to HDL size, total HDL particles, and total HDL lipids remained largely unchanged. HDL levels may be influenced by disease progression and medical interventions, such as lipid-lowering therapy initiated during follow-up [14,[50], [51], [52]]. Although adjustment for BMI and CVD would presumably indirectly account for use of lipid-lowering therapies to some extent, we cannot rule out presence of residual confounding.

In conclusion, this study found no association between HDL subfractions (particle size, concentration, and lipid composition), ApoA1, and the risk of a first-lifetime VTE. Our findings suggest that HDL may not play a role in the pathogenesis of VTE.

## Figures and Tables

**Supplementary Figure 6:**