# Supplementary material for: High-density lipoprotein subfractions and risk of future venous thromboembolism—the HUNT study
Source: Res Pract Thromb Haemost. 2025 Dec 9;10(1):103295. doi: 10.1016/j.rpth.2025.103295 (PMC12804113; doi:10.1016/j.rpth.2025.103295)
Supplement: Supplementary Material [file mmc1.docx]

Supplementary material

**Supplementary Table 1.** **Lipid distribution across HDL subfractions.** Mean concentrations (mmol/L) of HDL total particles and lipid components (phospholipids, total cholesterol, cholesteryl esters, free cholesterol and triglycerides), in small (S-HDL), medium (M-HDL), large (L-HDL) and very large (XL-HDL) HDL particles.

| **The mean concentrations (mmol/L) of total particles and lipid components across HDL subfractions** | |
| --- | --- |
| S-HDL |  |
| Total particles | 0.011 ± 0.001 |
| Phospholipids | 0.705 ± 0.094 |
| Total cholesterol | 0.500 ± 0.057 |
| Cholesteryl esters | 0.365 ± 0.045 |
| Free cholesterol | 0.135 ± 0.017 |
| Triglycerides | 0.060 ± 0.023 |
| M-HDL |  |
| Total particles | 0.004 ± 0.001 |
| Phospholipids | 0.515 ± 0.100 |
| Total cholesterol | 0.551 ± 0.120 |
| Cholesteryl esters | 0.448 ± 0.097 |
| Free cholesterol | 0.103 ± 0.025 |
| Triglycerides | 0.058 ± 0.026 |
| L-HDL |  |
| Total particles | 0.002 ± 0.001 |
| Phospholipids | 0.370 ± 0.163 |
| Total cholesterol | 0.378 ± 0.187 |
| Cholesteryl esters | 0.295 ± 0.146 |
| Free cholesterol | 0.083 ± 0.041 |
| Triglycerides | 0.035 ± 0.016 |
| XL-HDL |  |
| Total particles | 0.000 ± 0.000 |
| Phospholipids | 0.082 ± 0.049 |
| Total cholesterol | 0.097 ± 0.040 |
| Cholesteryl esters | 0.071 ± 0.032 |
| Free cholesterol | 0.026 ± 0.008 |
| Triglycerides | 0.009 ± 0.004 |

Concentrations are shown as mean ± standard deviation.

**Supplementary Figure 1. Relationship between HDL size and body mass index.** Scatter plot with a linear regression line illustrating the relationship between HDL Size (nm) and body mass index (kg/m^2^).

**Supplementary Figure 2.** Forest plot illustrating the hazard ratios (HRs) for venous thromboembolism (VTE) by high-density lipoprotein (HDL) particle characteristics in analysis restricted to the first 5-years of follow-up. HRs with 95% confidence interval (CI) are presented per one standard deviation increase in analysis adjusted for age, sex, and body mass index.

**Supplementary Figure 3. Quartile analysis of HDL particle ratios and VTE risk.** Forest plot presenting hazard ratios (HRs) of venous thromboembolism (VTE) by the ratio of very large high-density lipoprotein (HDL) (XL-HDL) to total HDL particles, and the combined ratio of very large (XL-HDL) and large (L-HDL) HDL particles. Quartile 1 (Q1) is the reference quartile. The analysis is adjusted for age, sex, and body mass index.

**Supplementary Figure 4. Lipid distribution across HDL subfractions.** Bar charts showing the mean concentrations (mmol/L) of lipid components (phospholipids, total cholesterol, cholesteryl esters, free cholesterol and triglycerides), in small (S-HDL, **4A**), medium (M-HDL, **4B**), large (L-HDL, **4C**) and very large (XL-HDL, **4D**) HDL particles. Error bars indicate 95% confidence intervals.

**Supplementary Figure 5. Relationship between ApoA1 and HDL particles. Figure 5A.** Scatter plot with a linear regression line illustrating the relationship between Apolipoprotein A1 (ApoA1) levels (g/L) and high-density lipoprotein (HDL) particle concentrations (mmol/L). **Figure 5B.** Forest plot of hazard ratios (HRs) with 95% confidence intervals (CIs) across quartiles (Q) of ApoA1 (g/L) and total HDL particles (mmol/L). Quartile 1 (Q1) is the reference quartile. The analysis is adjusted for age, sex, and body mass index.

**Supplementary Figure 6. Association between HDL particle characteristics and risk of unprovoked VTE.** Forest plot illustrating the hazard ratios (HRs) for venous thromboembolism (VTE) associated with high-density lipoprotein (HDL) particle characteristics, presented with 95% confidence interval (CI) per one standard deviation increase. The analysis includes three models. Model 1 is adjusted for age and sex. Model 2 is adjusted for age, sex, and body mass index (BMI). Model 3 is adjusted for age, sex, BMI, and cardiovascular disease (CVD).
